# Quantifying Potential Immortal Time Bias in Observational Studies in Acute Severe Infection

**DOI:** 10.1093/ofid/ofaf173

**Published:** 2025-03-18

**Authors:** Tom A Yates, Tom Parks, Peter J Dodd

**Affiliations:** Institute of Health Informatics, University College London, London, UK; Division of Infection and Immunity, University College London, London, UK; Department of Infectious Disease, Imperial College London, London, UK; Sheffield Centre for Health and Related Research, University of Sheffield, Sheffield, UK

**Keywords:** antimicrobials, clinical microbiology, clinical epidemiology, immortal time bias, infectious diseases, streptococcal toxic shock syndrome

## Abstract

**Background:**

Immortal time bias is a spurious or exaggerated protective association that commonly arises in naive analyses of observational data. It occurs when people receive the intervention because they survive, rather than survive because they received the intervention. Studies in conditions with substantial early mortality, such as acute severe infections, are particularly vulnerable.

**Methods:**

We developed IMMORTOOL, an R package accessible via a user-friendly web interface (https://petedodd.github.io/IMMORTOOL-live/). This tool will estimate the potential for immortal time bias using empiric or assumed data on the distributions of time to intervention and time to event. Assumptions are that no other biases are present and that the intervention does not affect the outcome. The tool was benchmarked using studies presenting both naive analyses and analyses with the intervention fit as a time-varying exposure. We applied IMMORTOOL to a set of influential observational studies that used naive analyses when estimating the impact of polyclonal intravenous immunoglobulin (IVIG) on survival in streptococcal toxic shock syndrome (STSS).

**Results:**

IMMORTOOL demonstrated that published estimates suggesting a survival advantage from giving IVIG in STSS are explained, at least in part, by immortal time bias.

**Conclusions:**

IMMORTOOL can quantify the potential for immortal time bias in observational analyses. Importantly, it simulates only bias resulting from misallocation of person-time, not other related selection biases. The tool may help readers interrogate published studies. We do not advocate IMMORTOOL being used to justify naive analyses where robust analyses are possible. To what extent giving IVIG in STSS improves survival remains uncertain.

## Teaser

Immortal time bias exaggerates estimates of treatment efficacy in naive analyses of observational data. We developed a tool to estimate the extent of this bias. The benefits of giving intravenous immunoglobulin in streptococcal toxic shock syndrome have likely been overstated.

Ethical and logistical considerations, including the regulatory burden placed on clinical trialists, mean that randomized controlled trials (RCTs) cannot address all important questions regarding the efficacy and safety of medical interventions. This is particularly true when the outcomes of interest are infrequent. Well-conducted observational studies, therefore, play an important role in informing clinical practice.

Patients may succumb to their disease before a treatment is initiated. In this scenario, observational studies that simply count deaths in the treated and untreated groups will overestimate the benefits of the intervention because patients need to survive long enough to receive treatment. Here, some of any “protective” association will result from people having received the intervention because they survived, as opposed to surviving because they received the intervention.

**Figure 1.**
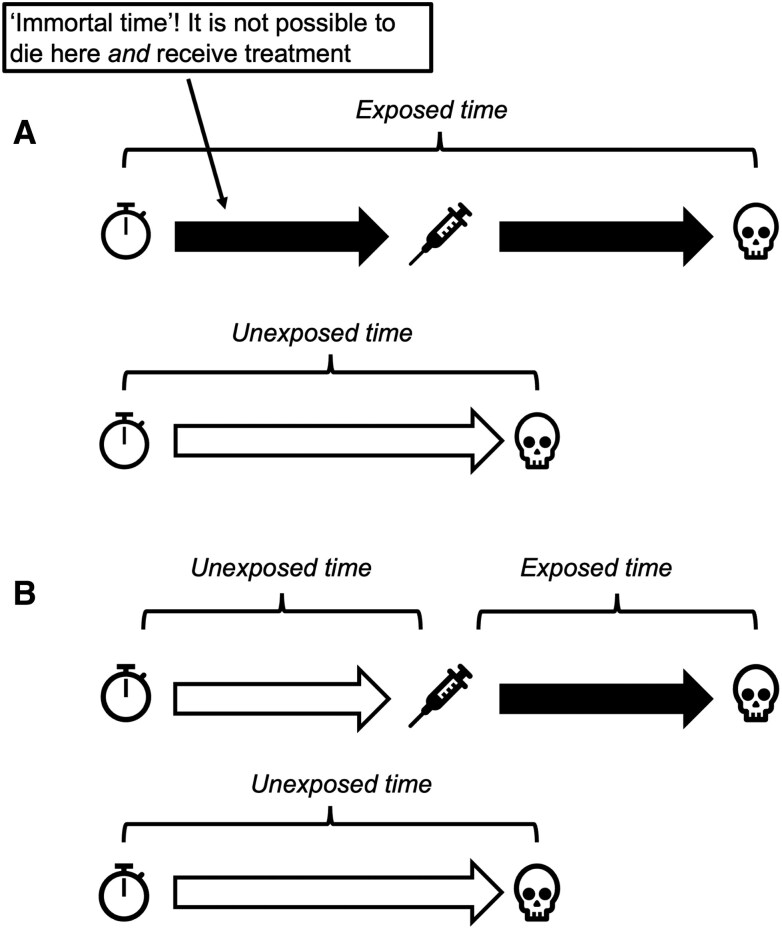
A schematic depicting the attribution of pre- and post-treatment time in the exposed and unexposed groups (*A*) applying a naive analysis and (*B*) with the intervention fit as a time-varying exposure.

This phenomenon is immortal time bias (ITB), a problem reportedly first described by William Farr in 1843 [1] and then rediscovered in the 1970s [2]. Pretreatment time in the treated group is misclassified because patients are yet to receive treatment (Figure 1). This time period is “immortal” because it is not possible to die prior to treatment and then receive treatment. Other terms for this type of survival bias include *time-dependent bias* and *survivor treatment selection bias*.

Naive analyses of this sort are commonly conducted and published despite there being methods available that result in person-time being properly allocated (Table 1) [3]. The 2 best approaches to avoiding misassignment of person-time are analyzing the intervention as a time-varying exposure and designing the study so that follow-up time in treated and untreated individuals is aligned.

**Table 1.** Methods Used to Address Misallocation of Person-Time

Solution	Advantages	Disadvantages
**Optimal solutions**		
Fit intervention as a time-varying exposure	Addresses misallocation of person-time	Requires granular data on both time to intervention and time to event Requires authors to have some statistical modeling skills Does not address related biases, such as prevalent user bias
Design study or analysis so that follow-up time in treated and untreated group are aligned [21, 25]	Addresses misallocation of person-time and related biases	Not always possible or straight forward
**Occasionally reasonable solutions**		
Landmark analysis (see text)	Computationally easy Can often be applied in data sets lacking granular data on time to intervention or time to event Addresses misallocation of person-time if appropriate landmark time chosen	Throws away data Produces estimates conditional on survival to landmark time, which may not be generalizable to individuals treated early Does not address related biases, such as prevalent user bias
**Problematic fixes**		
Exclude early events	Computationally easy Can often be applied in data sets lacking granular data on time to intervention or time to event May attenuate ITB	Some residual ITB is likely Throws away data Produces estimates conditional on survival to the time point chosen, which may not be generalizable to individuals treated early Does not address related biases, such as prevalent user bias
Exclude untreated individuals who die early	Computationally easy Can often be applied in data sets lacking granular data on time to intervention or time to event • May attenuate ITB	Throws away data Does not compare like with like, as treated patients who die early will be counted among the deaths—results may be hard to interpret • Does not address related biases, such as prevalent user bias

Abbreviation: ITB, immortal time bias.

Another approach, *landmark analysis*, is widely used [4]. Here, follow-up in the treated and untreated groups starts at a time by which most treatment has been administered (landmark time), with treatment status fixed at that point. This means that individuals who are subsequently treated will be counted among the untreated group. If an appropriate landmark time is chosen, misallocation of person-time is largely avoided. However, the approach is inefficient, disregarding information on events occurring prior to landmark time [4]. Furthermore, it generates estimates conditional on survival to landmark time. These estimates may not be generalizable to the overall population being considered for treatment, because the treatment decision is mostly taken at an earlier point in the disease process. For example, antibiotics may be less effective if administered late.

Various other, mostly problematic, “fixes” to address ITB are commonly applied (Table 1). Rather than being a consequence of ITB itself, the shortcomings of these approaches principally reflect the different treatment of exposed and unexposed groups. For example, time zero may not be aligned, or early deaths may be discounted in only 1 of the 2 groups. Such decisions mean that investigators are not comparing like with like—for example, comparing people at different stages of their illness or comparing survivors with an unselected group.

The extent to which ITB affects estimates of treatment effect will vary. If patients typically receive the intervention at a time point that is earlier than most observed deaths, then the bias will be small. While our focus here is mortality, ITB can also occur where other endpoints preclude receiving the intervention.

Here, we describe the design and validation of a simple tool that can be used to assess the extent to which misallocation of person-time might bias the results of observational studies that (1) do not use methods to account for immortal time or (2) use commonly applied but problematic methods, such as landmark analysis. While the tool can be applied to any observational study, the studies that motivated this work were observational analyses of interventions in acute severe infections where early mortality is high. Such analyses are particularly vulnerable to ITB, with naive analyses often generating results that suggest that interventions have implausibly large benefits [5].

Importantly, our tool addresses only bias resulting from misclassification of person-time. In our Conclusions section, we mention a set of related forms of selection bias that can occur despite the intervention being fit as a time-varying exposure.

## METHODS

### Description of the Tool

We developed an R package called IMMORTOOL, including a web browser interface, which will assess the potential for misallocation of person-time to affect study results. The tool can fit Weibull [6] mortality and exposure hazards using the data on cumulative outcomes by time that are typically reported in studies. The tool accounts for competition between mortality and exposure. Plots allow assessment of the plausibility of fits obtained. Under the assumption that the intervention has no effect, fitted hazards are then used to simulate a cohort with a default size of 100 000.

Apparent effect sizes are calculated as mortality rate ratios according to 4 commonly applied analytic approaches:

**(a) Person-time from time zero:** Pretreatment time in the treated group misclassified as “exposed” (our default and the most commonly applied naive approach).

**(b) Exclude early events and do not reset clock:** As above, but discounting deaths in both the treated and untreated groups that occur before a prespecified follow-up time, leaving time zero unchanged.

**(c) Exclude early events and reset clock:** As above, but discounting deaths in both the treated and untreated groups that occur before a prespecified follow-up time, and also resetting time zero to be that same time point.

**(d) Landmark analysis:** As above, but defining exposure status at the landmark time—that is, individuals treated after landmark time are retained in the untreated group.

These are defined formally in the supplementary appendix, with a graphic depicting all 4 approaches provided on the IMMORTOOL web interface. Some advantages and disadvantages of each are given in Table 1. For all approaches, the rate ratio is calculated as the ratio of total deaths over total person-time for those exposed vs unexposed. Finally, approximate confidence intervals are calculated by using the observed number of deaths and the expected fraction of deaths occurring in the exposed vs unexposed groups (see supplementary appendix).

### Benchmarking

To assess the performance of our tool, we used a set of infection-related observational studies that reported the same treatment effects using a naive approach and methods that properly allocate person-time [5, 7, 8]. Detailed descriptions of these studies can be found in the supplementary appendix.

Two of these studies undertook naive analyses but also fitted the intervention as a time-varying exposure. The first described mortality in people with *Staphylococcus aureus* bacteremia who had survived for 48 hours after the date of the first positive blood culture [5]. The analysis compared mortality in patients who did or did not receive a computed tomography–positron emission tomography (CT-PET) scan to screen for deep foci of infection. The second study described mortality in people admitted to intensive care beds with severe H1N1 influenza, comparing mortality in people who received or did not receive oseltamivir, an antiviral drug [7, 8]. The authors of this influenza study also provided a landmark analysis.

In both these studies, correct allocation of person-time substantially attenuated the protective association reported. It is important to note that observational analyses that properly allocate person-time may not estimate the true effects of the intervention, as confounding by indication and other biases may result in either an underestimate or an overestimate of the true association [9]. RCTs of both interventions are ongoing (NCT04381936, NCT02735707, NCT05137119).

The third study described the rate at which people admitted to the hospital with COVID-19 progressed to mechanical ventilation or death [10]. The analysis compared the incidence of this composite endpoint in people who did or did not receive the drug hydroxychloroquine. The authors presented a naive analysis and a landmark analysis. They did not include an analysis with the intervention analyzed as a time-varying exposure, but the true effects of the intervention are known from RCTs. This observational analysis and subsequent RCTs suggested that the intervention—hydroxychloroquine as a treatment for COVID-19—offered no benefit or was harmful [11].

It is important to be clear what should be expected from this benchmarking exercise. IMMORTOOL should identify the potential for substantial ITB where we know that it exists because the same data have been analyzed using naive methods and fitting the intervention as a time-varying exposure. This was the case with the CT-PET and influenza studies. However, naive results will not necessarily agree with IMMORTOOL predictions. IMMORTOOL assumes that the intervention has no impact on the outcome. It also assumes that no other biases are affecting results. Clearly, both assumptions may be unreasonable. Particularly in small data sets, the play of chance may also generate discrepancies.

### Application to a Clinical Problem: Intravenous Immunoglobulin in Streptococcal Toxic Shock Syndrome

We next used our tool to assess the likely impact of misallocation of person-time on observational studies in acute infection that did not model the intervention as a time-varying exposure. Here, our focus was on observational studies of polyspecific intravenous immunoglobulin (IVIG) in patients with streptococcal toxic shock syndrome (STSS).

We selected this example because STSS carries very high early mortality. IVIG is an expensive pooled blood product and, in many settings, not immediately available. As such, the risk of meaningful ITB seemed high. The only RCT of IVIG in STSS enrolled 18 patients and so cannot exclude either substantial benefit or substantial harm [12].

We took our data from a systematic review of observational studies describing mortality in patients with STSS who did or did not receive IVIG [13]. The review reported 30-day mortality in patients, all of whom had received adjunctive clindamycin. Data in this subgroup were not reported in all of the primary articles.

Three of these studies applied a naive analysis [14–16]. One study excluded untreated (but not treated) individuals who died within the first 12 hours [17]. We applied IMMORTOOL to the first 3 studies but not to the study excluding early deaths in the untreated. While excluding early deaths in the untreated might be expected to attenuate ITB, it comes at the cost of creating groups that are not directly comparable—that is, all treated individuals compared with untreated individuals who had survived for at least 12 hours. The net impact of this strategy is not something that IMMORTOOL is able to predict.

Only 1 of the remaining studies reported time to receipt of IVIG, stating that this was mostly initiated “during the first day of onset of illness” [16]. In our primary analysis, for all studies, we assumed that, in the treated group, 50% received the intervention within 12 hours. With only 1 time point—30-day mortality—provided in the clindamycin-treated STSS population, we used external data to parameterize the distribution of time to death [18]. In our primary analysis, we assumed that all deaths occurred within 8 days and that half of all deaths occurred within the first 48 hours. To emulate the original review, we pooled results using random effects meta-analysis.

In sensitivity analysis, we varied these assumptions to generate a “low ITB scenario” (50% of all IVIG given within 6 hours and 33% of all deaths occurring within the first 48 hours) and a “high ITB scenario” (50% of all IVIG given within 24 hours and 66% of all deaths occurring within the first 48 hours).

### Code and Tool Availability

We have made IMMORTOOL available as an R package, with a ShinyApp interface, so that readers can undertake their own analyses. The underlying code, including code to reproduce all results reported in this article, is available at https://github.com/petedodd/IMMORTOOL. Online access to the ShinyApp is available at https://petedodd.github.io/IMMORTOOL-live/. Note that, in some browsers, the interface will take a few minutes to load.

### Ethical Approvals

All analyses presented here used data in the public domain. Ethical approval to undertake these analyses was, therefore, not required.

## RESULTS

### Benchmarking

Results from the 3 articles that we used for benchmarking are presented in Table 2. Where possible, we present the results from the naive analysis as well as results treating the intervention as a time-varying exposure. Where articles presented crude and adjusted estimates, we present their primary adjusted estimates. These are presented alongside the estimates provided by IMMORTOOL. The IMMORTOOL results are an estimate of the intervention effect that might be expected purely as a result of misallocation of person-time, under the assumption that the intervention had no effect and that no other biases were at play.

**Table 2.** Published Results From the 3 Benchmarking Studies Alongside Estimates From IMMORTOOL

	Published Estimates (95% CI)			IMMORTOOL Estimates (95% CI)	
Study	Naive Approach	Landmark Approach	Intervention as Time-Dependent Variable	Naive Approach	Landmark Approach
van der Vaart [5]	aHR, 0.50 (.34–.74)	Not provided	aHR, 1.00 (.68–1.48)	RR, 0.60 (.42–.85)^a^	Not provided
Jones and Fowler [7]	HR, 0.52 (.29–.95)	OR, 0.39 (.23–.66)	HR, 0.87 (.48–1.61)	RR, 0.25 (.13–.47)^b^	RR, 1.00 (.66–1.5)^c^
Geleris [10]	aHR, 1.04 (.82–1.32)	aHR, 0.81 (.63–1.04)	Not provided^d^	RR, 0.49 (.40–.61)^b^	RR, 1.00 (.81–1.20)^c^

Abbreviations: aHR, adjusted hazard ratio; HR, hazard ratio; OR, odds ratio; RR, rate ratio.

^a^This used the IMMORTOOL approach (b) “Exclude early events and do not reset clock,” as van der Vaart excluded treated and untreated participants who died in the first 48 hours.

^b^This used the IMMORTOOL approach (a) “Person-time from time zero.”

^c^This used the IMMORTOOL approach (d) “Landmark analysis.”

^d^In randomized controlled trials, the RR is 1.09 (95% CI, .99–1.19) for all-cause mortality and 1.11 (95% CI, .91–1.37) for mechanical ventilation [11].

In the same table, we present the results of landmark analyses by Jones and Fowler [7] and Geleris et al [10] alongside estimates, applying the same landmark time, provided by IMMORTOOL. The IMMORTOOL results, again, represent an estimate of the intervention effect that might be expected purely as a result of residual misallocation of person-time, under the assumption that the intervention had no effect and that no other biases were at play.

IMMORTOOL was clearly able to identify the potential for substantial bias in naive analyses of the CT-PET and influenza studies, providing reassurance that the tool functions as intended. Further discussion of benchmarking results is provided below.

### IVIG in STSS

**Figure 2.**
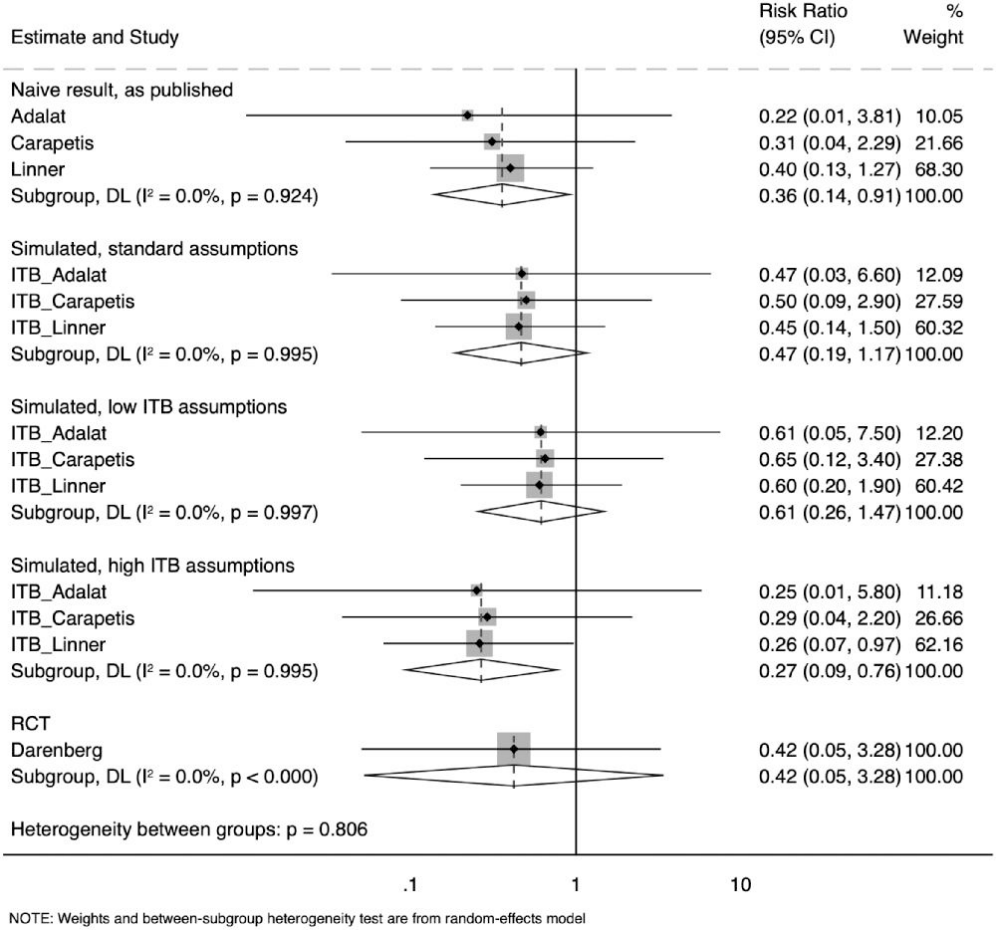
A forest plot containing the results reported by 3 observational studies estimating a risk ratio for 30-day mortality in patients with streptococcal toxic shock syndrome according to whether they received intravenous immunoglobulin; the results of 3 sets of IMMORTOOL simulations estimating the results expected as a result of misallocation of person-time, if the intervention has no effect on the outcome; and the results of the small randomized controlled trial (RCT). The standard simulation assumptions had 50% of those receiving the intervention receiving it within 12 hours and half of all deaths occurring within 48 hours. (Note: IMMORTOOL calculates a rate ratio rather than a risk ratio but, here, these measures should be broadly comparable.) The low immortal time bias (ITB) assumptions had 50% of those receiving the intervention receiving it within 6 hours and 33% of all deaths occurring within 48 hours. The high ITB assumptions had 50% of those receiving the intervention receiving it within 24 hours and 66% of all deaths occurring within 48 hours. DL, *DerSimonian and Laird*.

Estimates of the effects of IVIG on mortality in STSS are presented in Figure 2. All 3 observational studies [14–16] applied a naive analysis, and the data presented here are unadjusted for potential confounders. Also presented in Figure 2 are estimates from IMMORTOOL, under various assumptions about the distributions of time to event and time to intervention, plus the estimate from the single underpowered RCT [12]. These results suggest that misallocation of person-time in these analyses will have biased estimates of the benefits of IVIG in STSS away from the null. In a post hoc sensitivity analysis, findings were unchanged if the time-to-event distribution allowed for a small number of deaths after day 8.

## CONCLUSIONS

Inappropriate analyses of observational studies that fail to account for immortal time can result in the protective effects of interventions being overestimated. This is a particular risk in conditions with substantial early mortality, such as acute severe infections. By fitting to reported data and simulating a study with a true null effect, IMMORTOOL can calculate the likely extent of bias resulting from this misallocation of person-time. In doing so, it makes 2 key assumptions. The first—that the intervention has no effect on the outcome—is often true. The second—that no other biases are at play—is rarely true.

In the CT-PET and influenza studies [5], IMMORTOOL identified the potential for substantial ITB in the naive analyses, which was apparent when comparing these with the results fitting the intervention as a time-varying exposure. IMMORTOOL also suggested the potential for significant ITB in the hydroxychloroquine study [10]. That the naive estimate in that study suggested no benefit—in keeping with data from RCTs—may suggest that a second bias, most likely residual confounding by indication, offset the ITB. Here, we did not have results fitting the intervention as a time-varying exposure to directly compare with results from the naive analysis.

Clearly, other biases may attenuate or exaggerate naive effect estimates. In smaller studies, the play of chance will also result in naive estimates that do not align with those predicted by IMMORTOOL. Our benchmarking exercise does, however, suggest that the tool will highlight situations in which substantial ITB is likely.

The landmark analysis performed by the authors of the influenza study is instructive [8], as it resulted in an estimate of the treatment effect that was further from the estimate with the intervention fit as a time-varying exposure than the estimate from the naive analysis. This likely occurred because the approach throws away data, resulting in imprecision.

We used IMMORTOOL to interrogate 3 influential studies of the association between receipt of IVIG and 30-day mortality in STSS [14–16]. IMMORTOOL suggested that ITB was leading to exaggerated estimates of the benefits associated with IVIG treatment. Even where conservative assumptions were made regarding the distribution of time to intervention and time to event, IMMORTOOL estimated that misallocation of person-time might result in a rate ratio of 0.61 (95% CI, .26–1.47). While our results suggest that published estimates of the effects of IVIG on mortality in STSS overstate the survival advantage because of ITB, we caution against concluding that the intervention has no benefit. There are reasons that observational studies might underestimate the benefits of this intervention. For example, if IVIG were offered to the sickest patients with STSS, then a modest benefit might be masked by confounding by indication. Confounding by indication could also operate in the other direction. For example, IVIG administration might be associated with prompt STSS diagnosis and proactive management, and IVIG might be withheld from patients with a very poor prognosis, who are expected to do poorly regardless.

The most robust means of answering this important clinical question would be to undertake a large pragmatic multicenter RCT. The acuity of the situation, the potential harms associated with administering a blood product, and the significant limitations of the existing evidence base could argue in favor of deferred consent, as used in other recent trials in acute severe infection [19]. Deferred consent would likely improve enrollment. However, if the true benefits of IVIG are more modest than previously assumed, then this RCT would need to be large [20]. The only previous trial ceased recruitment after randomizing 18 participants because enrollment was too slow [12].

Our tool can assess the likely extent of bias due to misallocation of person-time in specific cases and includes the impact of sample size on precision. In smaller samples, exaggerated point estimates—in both directions—will be seen more commonly regardless of the extent of any bias. IMMORTOOL can also explore the impact of landmark analysis and of crude approaches to limit ITB, such as not counting deaths that occur early.

Importantly, ITB is related to other forms of selection bias, such as prevalent user bias, that are not fully addressed by fitting the intervention as a time-varying exposure (this only addresses misallocation of person-time). These biases occur where the intervention is harmful or protective—contrary to the assumption made by IMMORTOOL—and where treatment assignment and follow-up time are not aligned. Here, the most vulnerable individuals may be selectively depleted from the exposed or unexposed group such that, when follow-up begins, the 2 populations are not comparable. These issues and methods that can address selection problems beyond misallocation of person-time are discussed elsewhere [21].

A challenge in applying IMMORTOOL is that study authors often do not provide full data on the timings of the intervention or endpoints. However, readers will often have some idea of the expected distribution of time to death and can test a number of plausible time-to-intervention distributions.

It is noteworthy that reporting guidelines do not explicitly mandate reporting of the distribution of time to treatment or time to event in observational studies of health interventions. STROBE [22] requires authors to “report numbers of outcome events or summary measures over time,” but there is nothing further in the relevant STROBE extensions: RECORD [23] or RECORD-PE [24]. Updating these reporting guidelines to encourage the reporting of these distributions should be considered so that readers have the data required to assess risk of ITB.

It is not our intention that the tool be used to justify naive analyses where better analyses are possible. However, sometimes data do not allow for the intervention to be fit as a time-varying exposure. For example, to avoid misallocation of person-time in observational analyses, sufficiently granular data on time to intervention and time to event are required. Where interventions and outcomes occur over minutes or hours, analyses fitting time-to-intervention or time-to-event data in days or weeks may suffer from bias resulting from the misallocation of person-time. Here, investigators may find IMMORTOOL helpful in deciding whether to proceed with a naive analysis.

IMMORTOOL enables quantitative assessments of the likely extent of bias resulting from misallocation of person-time in published observational studies. This may flag studies where the published intervention effect is likely to be over optimistic. Alternatively, it may provide reassurance that such bias is unlikely, meaning that published results are not automatically disregarded, although clearly other forms of bias may still explain results. Where possible, important questions regarding the relative efficacy and safety of interventions should be answered in RCTs.

## Supplementary Material

ofaf173_Supplementary_Data

## Supplementary Data

Supplementary materials are available at *Open Forum Infectious Diseases* online. Consisting of data provided by the authors to benefit the reader, the posted materials are not copyedited and are the sole responsibility of the authors, so questions or comments should be addressed to the corresponding author.

## Notes

***Acknowledgments***. We are grateful to Dr Mark Jones and Prof Rob Fowler for answering our queries about their article exploring the impact of immortal time bias on estimates of the efficacy of oseltamivir in severe influenza.

***Patient consent***. As this was a secondary analysis of published data, patient consent was not required.

***Financial support.*** This work was supported by the National Institute for Health and Care Research (to T. A. Y. as an NIHR Clinical Lecturer); the Wellcome Trust (222098/Z/20/Z to T. P. as a Clinical Research Career Development Fellow); and the UK Medical Research Council (MR/P022081/1 a fellowship awarded to P.J.D.).

***Disclaimer.*** The views expressed in this publication are those of the authors and not necessarily those of the NHS, the National Institute for Health and Care Research, or the Department of Health and Social Care.
